# Mothers adapt their voice during children’s adolescent development

**DOI:** 10.1038/s41598-022-04863-2

**Published:** 2022-01-19

**Authors:** Simon Leipold, Daniel A. Abrams, Vinod Menon

**Affiliations:** 1 Stanford Cognitive and Systems Neuroscience Laboratory, Department of Psychiatry and Behavioral Sciences, Stanford University, 401 Quarry Rd., Stanford, CA 94305 USA; 2 Department of Neurology and Neurological Sciences, Stanford University, Stanford, USA; 3 Stanford Neurosciences Institute, Stanford University, Stanford, USA

**Keywords:** Psychology, Human behaviour

## Abstract

Mothers alter their speech in a stereotypical manner when addressing infants using high pitch, a wide pitch range, and distinct timbral features. Mothers reduce their vocal pitch after early childhood; however, it is not known whether mother’s voice changes through adolescence as children become increasingly independent from their parents. Here we investigate the vocal acoustics of 50 mothers of older children (ages 7–16) to determine: (1) whether pitch changes associated with child-directed speech decrease with age; (2) whether other acoustical features associated with child-directed speech change with age; and, (3) the relative contribution of acoustical features in predicting child’s age. Results reveal that mothers of older children used lower pitched voices than mothers of younger children, and mother’s voice pitch height predicted their child’s age. Crucially, these effects were present after controlling for mother’s age, accounting for aging-related pitch reductions. Brightness, a timbral feature correlated with pitch height, also showed an inverse relation with child’s age but did not improve prediction of child’s age beyond that accounted for by pitch height. Other acoustic features did not predict child age. Findings suggest that mother’s voice adapts to match their child’s developmental progression into adolescence and this adaptation is independent of mother’s age.

## Introduction

Mother’s voice is a highly salient social stimulus from the beginning of a child’s life. During prenatal development, fetuses are able to discriminate their mother’s voice from an unfamiliar female voice^1^, and, shortly after birth, newborns will actively exert effort to hear their mother’s voice^2^. During later stages of childhood, mother’s voice plays a foundational role in their child’s social-emotional^3^ and language development^4^. Neuroscience research has shown that hearing brief samples of mother’s voice elicits increased activity in a wide range of brain systems associated with salience, reward, and emotion processing in school-aged children^5^. These findings establish mother’s voice as a critical biological signal for children and highlight the centrality of this signal for multiple aspects of child development.

When speaking to a child, mothers and caregivers modify their voice in a stereotypical manner^6,7^. This unique manner of speaking is called infant-directed speech or child-directed speech, sometimes known as *baby talk* or *motherese*^8^. Child-directed speech contains simple, redundant utterances, spoken in a slow tempo with long pauses^9^. Furthermore, it is characterized by acoustical features such as a high pitch, a wide pitch range, and a short duration of utterances^10^, as well as specific timbral features^11^. Regarding the possible functions of child-directed speech, three main hypotheses have been proposed^12^, for a review see^13^. First, child-directed speech is thought to capture and maintain the child’s attention. It might be a particularly perceptually salient stimulus for a child, and thus, child-directed speech might effectively engage the child’s interest^10^. Second, child-directed speech might be used to communicate (positive) emotions to the child^14^. Third, caregivers might adapt their speaking style to teach children about language, by exaggerating certain linguistic aspects of speech to facilitate learning^15,16^.

The three hypothesized functions are not static over the course of childhood: Child-directed speech is thought to serve different functions during different phases of a child’s development to meet the needs of the child^17–19^. For example, Fernald^17^ proposed that during the first months of a child’s life, the attentional and emotional functions of child-directed speech are particularly important for regulating the child’s emotional state because the child is still in a stage where she is not yet able to produce meaningful utterances. Towards the end of the first year, once the child starts to utter her first words, the linguistic functions facilitating the child’s language acquisition become most relevant. Empirical evidence supporting these dynamics comes from a recent longitudinal study demonstrating that linguistic modifications in speech addressed to children aged 9 months and older, but not before, predicts the child’s vocabulary size at age 15 and 19 months^18^. Further modifications related to attentional and emotional functions of child-directed speech did not predict vocabulary size^18^.

Consistent with the fluid nature of parent–child interactions over time, the acoustical signature of mother’s voice dynamically changes throughout early stages of childhood^20^. For example, previous studies of mother’s voice have shown that maternal pitch height increases during the first 4 to 6 months of a child’s life before decreasing in a linear fashion^20,21^. Pitch height reflects the mean fundamental frequency (F0) of the vocal-acoustical signal^22^. Pitch height has been associated with capture and maintenance of attention in child-directed speech^23–25^. Thus, a decrease in pitch height is thought to reflect a change in mother–child interaction from a face-to-face setting focused on keeping the child alert and interested towards an actor-observer setting in which the child explores her immediate environment while relying on the mother to facilitate the child’s explorations, thereby shifting the focus of attention of the child away from the mother^20^.

However, it is unclear how long maternal pitch height continues to decrease with age: One study concluded that maternal pitch height stabilizes, reaching a pitch height comparable to adult-directed speech, before the child is 5 years old^26^, while a second study reported that maternal pitch height continues to be higher when speaking to 5-year-old children, and this pitch height is intermediate between infant-directed speech, which has the highest pitch heights, and adult-directed speech^27^. To our knowledge, previous studies have not investigated acoustical attributes of mother’s voice for children older than 5 years of age. This is an important question since a mother’s voice continues to be a constant presence in the lives of older children and adolescents, and it is unknown whether mothers make vocal accommodations to aid in communication with their child beyond the early childhood years. It is a particularly interesting question in the context of adolescence, a transformative period of development during which children’s cognitive and social behaviors become more adultlike and independent^28^.

### Aims of the study

The overall goal of the current study was to examine acoustical changes in maternal voice in older children and adolescents. Following the assumption that adaptations in mother’s voice match a child’s developmental needs, we hypothesized that the transition of a child from late childhood to early adolescence would be reflected in the vocal acoustical signature of a mother. We analyzed brief samples of 50 mothers’ voices in relation to the age of their children, who were between 7 and 16 years of age. We investigated whether maternal vocal features were correlated with, and predictive of, the child’s age, with three primary aims for the analysis: For the first aim, we focused on pitch height, the most salient acoustical feature of mother’s voice^25^. Based on earlier studies suggesting that maternal pitch is negatively correlated with the child’s age for most of early childhood before age 5^26^, we hypothesized that pitch height of mother’s voice would continue to decrease during late childhood and early adolescence. An important consideration for our analysis is that adolescents tend to have older mothers compared to younger children, and studies have shown that, for female participants, vocal pitch height decreases as a function of age over the lifetime^29,30^. Therefore, we assessed the effects of mother’s age on the hypothesized relationship between maternal pitch height and their child’s age. Crucially, we tested if pitch height of mother’s voice would decrease as a function of their child’s age *independent* of mother’s age. Second, we focused on additional acoustical features of mother’s voice that have been assessed in previous studies of child-directed speech, including pitch range, duration, amplitude, and brightness, a timbral feature, and assessed their power in predicting child’s age. Brightness describes an attribute of a sound that is perceived by a listener as part of the timbre and primarily reflects the high frequency content of the vocal-acoustical signal^31^. Third, to assess the importance of each acoustical feature in predicting the child’s age during late childhood and early adolescence, we quantified the relative contribution of each acoustical feature to the prediction, considering multiple features simultaneously.

## Materials and methods

### Participants

Mother–child dyads were recruited in the context of a brain imaging study investigating the neural processing of mother’s voice in children^5,32^. Here, we focus on the acoustical characteristics of vocal samples provided by the mothers. Mother–child dyads were recruited from around the San Francisco Bay Area in California, USA, for this study. Recruitment was performed through the internet, fliers sent to schools and posted at libraries, and word of mouth referrals.

We analyzed the vocal acoustics of 50 mothers whose children were between 7.72 and 16.47 years of age (n = 18 girls, n = 32 boys). With a sample size of n = 50, we had 80% statistical power to detect medium to large effects, according to Cohen^33^, in a Pearson correlation test setting. Mothers had a mean age of 46.46 years (standard deviation = 7.35 years, range 28.17–68.17 years). They had between one and three children at the time of the study (mean = 1.97 children, standard deviation = 0.73), including the child participating in the study. Children were the biological offspring of the mothers (i.e., none of the children were adopted, and therefore none of the mothers’ voices were from an adoptive mother), and all participants were raised in homes that included their mothers. The mother–child dyads lived in a wide range of socioeconomic backgrounds, with 18.7% of dyads coming from households earning ≤ $100,000 per year. The range of gross annual household income was $20,000 to $200,000 and over. N = 48 mothers communicated with their children primarily in English and n = 2 mothers spoke with their children mostly in Spanish. Mothers reported normal hearing, no personal or family (first degree) history of developmental cognitive disorders or neuropsychiatric disorders; no evidence of significant difficulty during pregnancy, labor, delivery, or the immediate neonatal period; and no abnormal developmental milestones as determined by neurologic history and examination of the children.

### Ethics statement

The Stanford University Institutional Review Board approved the study protocol, and the study was carried out in accordance with all relevant guidelines and regulations. Informed consent was obtained for all evaluation procedures from all participating children and mothers, and children were compensated for their participation in the study.

### Voice recordings

Mother’s voice samples were recorded in a quiet conference room using a Shure PG27-USB condenser microphone (Shure Inc., Niles, IL, USA) connected to a MacBook Air computer (Apple Inc., Cupertino, CA, USA). The audio signal was digitized at a sampling rate of 44.1 kHz and was A/D converted with 16-bit resolution. Mothers were seated in a position in a conference room to avoid early sound wave reflections from contaminating the voice recordings. The mouth-to-microphone distance was approximately 15 to 20 cm (6 to 8 inches) for all participants, and the input level of the microphone on the recording computer was set to the same level for all vocal recordings. The child was undergoing neuropsychological testing in a separate room during the recordings. Importantly, mothers were told that their voice recording would be played back to their child during the brain imaging experiment so that they could be attuned for “child-directed speech” even though children were not present in the recording session. Moreover, previous studies of child-directed speech have demonstrated that simulated child-directed speech, such as that used in the current voice recording protocol, has the same acoustical alterations compared to when children are present, although to a lesser extent^34–36^.

To provide a natural speech context, mothers were asked to repeat three sentences, each of which included a nonsense word. The first word of each of these sentences was their child’s first name, which was followed by the words “that is a”, followed by one of three nonsense words: “teebudishawlt”, “keebudishawlt”, or “peebudishawlt”. A hypothetical example of a sentence spoken by a mother for the recording was “Danielle, that is a peebudishawlt”. The inclusion of the child’s first name as the initial word in the sentence was an effort to keep mothers focused on their child who was participating in the brain imaging experiment and preventing mothers from thinking of other children or people when producing the sentence. Before the start of the recordings for each nonsense word, mothers were trained on how to produce the nonsense word. First, mothers were presented with a phonetic spelling of the nonsense word. Next, the experimenter (i.e., lab member) produced the nonsense word for the mother several times, and then the mother was asked to produce the nonsense word for the experimenter several times until they had reached proficiency. If the mother did not produce the word correctly, the experimenter corrected their pronunciation and asked them to produce it again. The experimenter did not begin recording the mother’s voice until the mother could easily and consistently produce the nonsense word correctly. The vast majority of mothers had very little difficulty producing any of the nonsense words and were therefore able to focus their attention on producing child-directed speech. A small number of the mothers had difficulty learning how to pronounce the nonsense words. In these cases, the experimenter would identify the part of the nonsense word that the mother was mispronouncing and provide feedback to the mother on how to pronounce it correctly. This process would continue until the mother was able to say the nonsense words correctly five times in a row. Mothers were instructed to say these sentences using the tone of voice they would use when speaking with their child during an engaging and enjoyable shared learning experience (e.g., if their child asked them to identify an item at a museum). The vocal recording session resulted in digitized recordings of the mothers repeating each of the three sentences about 30 times to ensure multiple high-quality samples of each nonsense word for each mother.

### Vocal sample postprocessing

The goal of vocal sample postprocessing was to isolate the three nonsense words from the sentences that each mother spoke during the recording session for acoustical analysis. We focused on the nonsense words because these words lack meaning, allowing us to study acoustical features of mother’s voice independent of semantic content. We used Audacity (RRID:SCR_007198), a digital sound editor, to isolate each utterance of the three nonsense words from the sentences spoken by each mother. The three best versions of each nonsense word were selected based on the audio and vocal quality of the utterances (i.e., eliminating versions that were mispronounced, included vocal creak, or were otherwise not ideal exemplars of the nonsense words).

### Acoustical analysis

**Figure 1:**
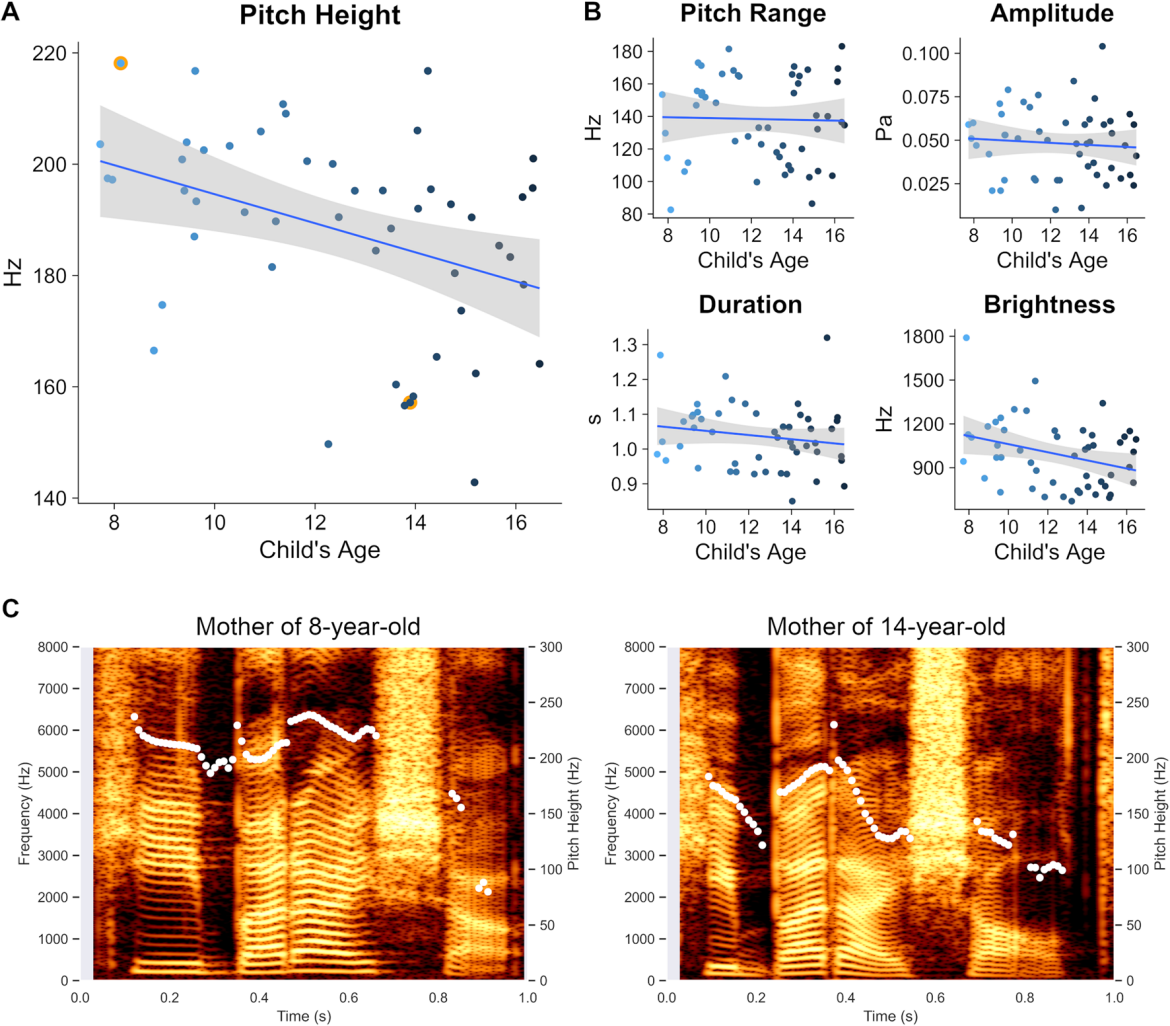
Relation between acoustical features of mother’s voice and child’s age. **(A)** Pitch height measured in the mother’s voice samples was negatively correlated with their child’s age (*r* =  − 0.38, *p* = 0.007). Data points for which spectrograms are visualized in **(C)** are highlighted in orange color. **(B)** Brightness, an acoustical feature which is positively correlated with pitch height, also showed a statistically significant association with the child’s age (r =  − 0.32, *p* = 0.02). However, multiple linear regression models including all acoustical features simultaneously showed that maternal pitch height was the only statistically significant predictor of child’s age, when the associations of all other acoustical features with child’s age were controlled for. Inclusion of brightness did not improve the prediction of child’s age, compared to a model including solely maternal pitch height. None of the other acoustical features of mother’s voice, including pitch range, amplitude, and duration, showed a statistically significant association with the child’s age (*p* > 0.20 for all correlations). **(C)** Spectrograms of two representative mothers articulating the nonsense word “keebudishawlt”. F0 values quantifying maternal pitch height are shown in white (right-sided y-axis). Brighter colors represent higher power in the time–frequency domain (left-sided y-axis).

Acoustical properties of the selected voice samples were analyzed using *Parselmouth* (version 0.3.3)^37^ in Python (RRID:SCR_008394; version 3.8.2). Parselmouth is a Python library for Praat software (RRID:SCR_016564). The following acoustical features were derived from each of the nine samples for every mother: pitch height, pitch range, duration, amplitude, and brightness. Feature selection was guided by both seminal and recent studies from the literature on the acoustical signature of maternal child-directed speech^10,11,25^. We used default settings for feature extraction unless otherwise stated. To calculate pitch height, we extracted the F0 for every 10 ms of each sample using the *to_pitch* command (pitch floor = 75 Hz, pitch ceiling = 300 Hz)^38^. This resulted in a timeseries of F0-values that was subsequently averaged per sample. The quality of pitch height extraction was visually inspected using spectrograms as in Fig. 1C. Pitch range was calculated subtracting the minimum F0 from the maximum F0 in the timeseries of F0 values, separately for each sample. Duration was obtained using the *get_total_duration* command. Vocal amplitude was quantified by the root mean square amplitude using the *get_rms* command. Finally, brightness was inferred based on the spectral centroid (center of mass) of each sample’s audio signal in the frequency domain using the *get_center_of_gravity* command. These analyses yielded one value for each of the acoustical features per sample, which were then entered into statistical analysis.

Control analyses were performed to examine the robustness of the primary results. To examine whether drop-off in pitch height at the end of the four syllable nonsense words influenced the results, only the highest pitches within the timeseries of F0-values of each voice sample (90th percentile) were computed for statistical analysis. To further examine this question, F0-values from the first 500 ms of each voice sample, encompassing the first syllables of the nonsense words, were computed for statistical analysis.

### Statistical analysis

Statistical analyses were performed in R (version 3.6.3, RRID:SCR_001905). Cross-validation analysis was performed using MATLAB (R2020a, RRID:SCR_001622). The statistical significance level was set to α = 0.05. First, we evaluated the association between mother’s pitch height and the child’s age using the Pearson correlation coefficient *r*. For this, we averaged the pitch height values across the nine voice samples to obtain one value per mother. To confirm the robustness of the association, we estimated *r*_(observed, predicted)_, a cross-validated measure of how well the independent variable (pitch height) predicts the dependent variable (child’s age), using a repeated 4-fold cross-validation (100 iterations). Data points were randomly assigned to four folds, with the constraint that the mean values of both the independent and dependent variable did not differ across folds; this constraint was implemented by repeating the random assignment as necessary until there was no evidence for differences between folds (*p* > 0.50), according to a one-way ANOVA. Then, a linear model was built using three folds, leaving out the fourth, and this model was used to predict the data in the omitted fold. This procedure was repeated four times leaving out each fold once. Finally, the statistical significance of the model was assessed using a permutation test. The empirical null distribution of *r*_(observed, predicted)_ was estimated by generating 1000 surrogate datasets under the null hypothesis of no association between mother’s pitch height and child’s age^39^. Next, we tested if the hypothesized association between mother’s pitch and child’s age was confounded by mother’s age. We evaluated the association between mother’s pitch height and the child’s age, controlling for mother’s age, using partial correlation analyses. The partial correlation between mother’s pitch height and child’s age was calculated by fitting linear models, separately for mother’s pitch height and child’s age as the dependent variables, using mother’s age as the independent variable. We extracted the residuals of these linear models and calculated the Pearson correlation coefficient *r* between the residuals. The partial correlation coefficient *r*_partial_ describes the linear association between mother’s voice and the child’s age, controlling for mother’s age.

Second, we assessed if acoustical features of mother’s voice other than pitch were associated with the child’s age, again using Pearson’s *r*. As with pitch height, we averaged the respective values across the nine samples for each mother, separately for pitch range, duration, amplitude, and brightness. A weakness of taking the mean across the nine voice samples is that this approach does not explicitly consider variance in the acoustical features across different utterances within each mother. Thus, we repeated the assessment of the associations between acoustical features and age without averaging across samples using linear mixed effects models which included a random intercept for each participant. Note that a random intercept model is needed to model the dependent vocal samples from each participant. Each mother provided nine samples and these samples of one mother were, on average, more similar compared with samples from another mother. We ran separate models for each acoustical feature. Mixed effects models were run using the R packages *lme4* (RRID:SCR_015654) and *lmerTest* (RRID:SCR_015656). To quantify the association between an acoustical feature and child’s age, we report standardized betas (β_std_), calculated using the R package *effectsize*. For brightness, a timbral feature, we included a main effect of nonsense word (“teebudishawlt”, “keebudishawlt”, or “peebudishawlt”) and an interaction effect between word and age to the model. Main and interaction effects for nonsense word were included for brightness because the initial consonant of the nonsense words, /t/, /k/, and /p/, are stop consonants which are characterized by distinct high-frequency content in the spectra of the audio signal^40^. The differential high-frequency content directly affects the spectral centroid of the vocal-acoustical signal, which was our acoustical proxy for brightness. Third, we inferred the relative contribution of each acoustical feature in predicting child’s age using multiple linear regressions with the acoustical features as predictors and the child’s age as the dependent variable. Finally, we ran a number of control analyses to determine the robustness of our findings with regard to the language mothers primarily used with their children (English or Spanish), potentially differential effects for mothers of girls vs. boys, potential effects of pitch height drop-off within the nonsense words, and potential effects of the chosen pitch extraction parameters. For all analyses, we report the lower and the upper boundary of the 95% confidence interval (CI) for the parameter of interest (i.e. *r* or β_std_).

## Results

The first aim of our study was to determine whether pitch height of mother’s voice changes with age during children’s late childhood and early adolescence. The average pitch height across all participants was 188.11 Hz (standard deviation = 18.20 Hz). As shown in Fig. 1A, results indicated a negative correlation of medium effect size between child’s age and pitch height of their mother’s voice (*r* = − 0.38, 95% CI [− 0.60, −  0.11], *t*(48) =  − 2.85, *p* = 0.007). This effect was statistically significant even after family-wise error (FWE) correction of the *p* value across the five investigated acoustical features (*p*_FWE_ = 0.035). 4-fold cross-validation analysis confirmed the robustness of this association, *r*_(observed, predicted)_ = 0.34, *p* = 0.006. Partial correlation analyses controlling for the effect of mother’s age corroborated the association between children’s age and pitch height of mother’s voice (*r*_partial_ =  − 0.33, 95% CI [− 0.56, − 0.06], *t*(48) =  − 2.41, *p* = 0.02). Cross-validation analysis controlling for mother’s age (using the residuals of child’s age and maternal pitch height after regressing out mother’s age) again confirmed the robustness of the association, *r*_(observed, predicted)_ = 0.27, *p* = 0.02. Mother’s age and child’s age were positively correlated (*r* = 0.38, 95% CI [0.11, 0.59], *t*(48) = 2.81, *p* = 0.007), but there was no significant relation between mother’s age and mother’s pitch height (*r* =  − 0.22, 95% CI [− 0.47, 0.06], *t*(48) =  − 1.57, *p* = 0.12). Thus, mother’s voice pitch decreases as a function of their child’s age in children and adolescents, and this acoustical cue is sufficient to predict their child’s age. Crucially, both of these effects persisted after accounting for mother’s age. Figure 1C shows the spectrograms and F0 timeseries for two representative mothers of a younger and an older child, demonstrating the observed effects.

The second aim of our study was to determine the relationship between child’s age and four other acoustical features of their mother’s voice (Fig. 1B). Results revealed no statistical evidence for an association between child’s age and maternal pitch range (*r* =  − 0.03, 95% CI [− 0.30, 0.25], *t*(48) =  − 0.18, *p* = 0.86), duration (*r* =  − 0.17, 95% CI [− 0.43, 0.11], *t*(48) =  − 1.21, *p* = 0.23), or amplitude (*r* =  − 0.08, 95% CI [− 0.35, 0.20], *t*(48) =  − 0.54, *p* = 0.59). On the contrary, we found evidence for a negative correlation of child’s age and brightness of mother’s voice (*r* =  − 0.32, 95% CI [− 0.55, − 0.04], *t*(48) =  − 2.33, *p* = 0.02). Brightness and pitch height of mother’s voice were positively correlated (*r* = 0.34, 95% CI [0.07, 0.57], *t*(48) = 2.52, *p* = 0.02).

To further examine the relationship between a child’s age and acoustical features of their mother’s voice, additional analyses were performed using linear mixed effects models, which consider the variance across the nine vocal samples measured for each mother. Results from this analysis yielded similar results: Again, we found a statistically significant association between child’s age and maternal pitch height (β_std_ =  − 0.32, 95% CI [− 0.54, − 0.11], *t*(50) =  − 2.90, *p* = 0.005), but no statistical evidence for an association between child’s age and pitch range (β_std_ =  − 0.02, 95% CI [− 0.18, 0.15], *t*(50) =  − 0.18, *p* = 0.86), duration (β_std_ =  − 0.15, 95% CI [− 0.39, 0.09], *t*(50) =  − 1.23, *p* = 0.22), or amplitude (β_std_ =  − 0.07, 95% CI [− 0.31, 0.18], *t*(50) =  − 0.53, *p* = 0.60). We found a negative association between child’s age and brightness in mother’s voice when not considering the influence of the initial consonant of the nonsense word (β_std_ =  − 0.26, 95% CI [− 0.47, − 0.05], *t*(50) =  − 2.37, *p* = 0.02), however, including an effect of nonsense word resulted in a non-significant association between brightness and child’s age (main effect: β_std_ =  − 0.19, 95% CI [− 0.42, 0.03], *t*(60.83) =  − 1.67, *p* = 0.10). We found a significant interaction effect between brightness of nonsense word and age which suggests that the relationship between brightness and child’s age was not consistent across the three different nonsense words but mostly driven by the nonsense word “keebudishawlt” (interaction effect: β_std_ =  − 0.13, 95% CI [− 0.25, − 0.01], *t*(400) =  − 2.14, *p* = 0.03). This interaction result suggests reduced generalizability of the association between brightness of mother’s voice and child’s age. Figure S1 in the supplemental material shows the relations between child’s age and acoustical features considering the variance of acoustical features between the separate vocal samples of each mother. For brightness, the figure includes separate regression lines for each of the three nonsense words, visualizing the interaction between age and nonsense word.

The third aim of our study was to assess the importance of each acoustical feature in predicting the child’s age during late childhood and early adolescence using a multiple linear regression model that included all acoustical features simultaneously (i.e. simultaneous multiple regression). Results revealed that maternal pitch height was the only statistically significant predictor of child’s age, even when the associations of all other acoustical features with child’s age were controlled for (see Table 1 for details).

**Table 1:** Full linear model of mother’s voice acoustics predicting child’s age.

Predictor	β_std_	95% CI_lower_	95% CI_upper_	*t*	*p*
Pitch height	− 0.40	− 0.73	− 0.07	− 2.46	0.02*
Pitch range	0.15	− 0.15	0.46	1.01	0.32
Duration	− 0.05	− 0.34	0.24	− 0.37	0.72
Amplitude	0.07	− 0.23	0.38	0.48	0.64
Brightness	− 0.20	− 0.50	0.11	− 1.30	0.20

*Statistically significant at α = 0.05; *β*_*std*_ standardized beta, *CI* confidence interval. The table provides statistical values for a simultaneous multiple regression, in which all acoustical features are entered into the regression model at the same time. The standardized betas for each acoustical feature are interpreted in the context of the influence of all the other acoustical features.

Inclusion of other acoustical features of mother’s voice did not improve the prediction of child’s age, compared to a model including solely maternal pitch height (see Table 2 for details on the stepwise multiple regression). These results indicate that the pitch height of mother’s voice is the exclusive contributor in predicting child’s age.

**Table 2:** Model comparisons.

Model	Adjusted R-squared	Δ Adjusted R-squared	F	*p*
Pitch height	0.13		(8.10)^#^	(0.007*)^#^
Pitch height + pitch range	0.14	0.01	1.39	0.24
Pitch height + duration	0.12	− 0.01	0.74	0.39
Pitch height + amplitude	0.12	− 0.01	0.43	0.52
Pitch height + brightness	0.15	0.02	2.32	0.13

*Statistically significant at α = 0.05.

^#^Compared to a null model (only including an intercept). The table provides statistical values for a stepwise multiple regression, in which one acoustical feature at a time is entered into the regression model. The first row (*Pitch Height*) shows results of statistical analysis for a model that includes only pitch height as a predictor of child’s age. Pitch height is a statistically significant predictor of child’s age as indicated by the *p* value in the first row. The subsequent rows (*Pitch Height* + *Pitch Range* through *Pitch Height* + *Brightness*) provide statistical values for the improvement of the model when the respective acoustical feature (e.g., Pitch Range in the second row) is included, compared to the Pitch Height-only model. For example, *Pitch Height* + *Pitch Range* did not explain significantly more variance in child age than Pitch Height alone as indicated by the *p* value in the second row.

Finally, we conducted control analyses to test the robustness of our findings: (i) Excluding mothers who primarily spoke Spanish with their children (n = 2) did not affect the magnitude or statistical significance of the relationship between mother’s pitch height and child’s age (*r* =  − 0.37, 95% CI [− 0.59, − 0.09], *t*(46) =  − 2.71, *p* = 0.009). (ii) When separately analyzing mothers of girls (n = 18) and mothers of boys (n = 32), the trend of a negative relationship between pitch height and child’s age was present for both mothers of girls (*r* =  − 0.49, 95% CI [− 0.78, − 0.03]) and mothers of boys (*r* =  − 0.30, 95% CI [− 0.59, 0.05]). (iii) Our participants spoke nonsense words consisting of four syllables, leading to a drop-off in pitch height towards the end of the utterances (c.f. Fig. 1C). To ensure this drop-off did not influence the association between pitch height of mother’s voice and child’s age, we analyzed only the highest pitches within the timeseries of F0-values of each voice sample (90th percentile), which confirmed that drop-off did not influence the negative relationship between pitch height and child’s age (*r* =  − 0.41, 95% CI [− 0.62, − 0.15], *t*(48) =  − 3.10, *p* = 0.003). The same was true when only analyzing the F0-values of the first 500 ms of each voice sample encompassing the first syllables of the nonsense words where no drop-off is present (*r* =  − 0.39, 95% CI [− 0.60, − 0.13], *t*(48) =  − 2.95, *p* = 0.005). (iv) Pitch excursions in infant-directed speech may go up to 400–500 Hz and higher on occasion^10^. To ensure our chosen pitch ceiling did not affect the association between pitch height of mother’s voice and child’s age, we additionally calculated pitch height using a ceiling parameter of 550 Hz. This reanalysis confirmed that pitch ceiling did not change the negative relation between mother’s vocal pitch and child’s age (*r* =  − 0.33, 95% CI [− 0.56, − 0.06], *t*(48) =  − 2.41, *p* = 0.02). Taken together, maternal pitch height showed a robust negative association with child’s age regardless of certain methodological choices.

## Discussion

Mothers alter multiple aspects of their voice, including increasing pitch height, when communicating with their young children (< 5 years old), however little is known regarding the acoustical features of mother’s voice for older children and adolescents. Here we show a reduction in the pitch of mother’s voice across a wide age range of children and adolescents (7–16 years old), and that the pitch height of mother’s voice is sufficient to predict their child’s age. Crucially, the association between maternal pitch height and child’s age was independent of effects of mother’s age on pitch height. Brightness, a timbral feature which is positively correlated with pitch height, also showed an inverse relation with child’s age. However, the inclusion of brightness did not improve prediction of child’s age over and above pitch height. In a regression model considering all acoustical features simultaneously, no other acoustical features of mother’s voice showed a significant relationship with child’s age, highlighting the specificity of pitch height as the primary acoustical feature that mothers consistently alter during child-communication across this age range. Results suggest that mothers make vocal compensations when speaking with their child across an extended period of development, reflecting the dynamic nature of mother–child interactions.

Child-directed speech is characterized by a range of pitch, durational, and timbral features^9–11,13^, and the primary goal of our study was to identify acoustical signatures of mother’s voice that vary across middle childhood and adolescence. Our analysis revealed that mother’s vocal pitch height decreased as a function of child’s age and this effect was independent of mother’s age. Previous studies investigating acoustical changes in mother’s voice as a function of child’s age have focused on young children (neonates to 5 years of age). These studies have shown that maternal pitch height increases during the first months after birth, peaks at 4 months, and then linearly decreases until age 2^20,21^. However based on the previous literature it is unclear whether pitch height has stabilized by the time the child reaches school age^26^, or not^27^. Our results add to this literature by providing evidence that a decrease in pitch height of mother’s voice continues throughout late childhood and adolescence. Furthermore, we showed that none of the other acoustical features examined in our analysis reveal this relationship with child’s age after controlling for pitch height. The specificity of mother’s voice pitch height effects in our study is consistent with findings from a seminal study showing that infants specifically prefer pitch-based modifications in child-directed speech^25^. Thus, compared with other acoustical features, including pitch range or the amplitude and duration of utterances, results are consistent with the hypothesis that maternal pitch height occupies a special role in the age-related dynamics of child-directed speech. An exciting avenue for future research is examining these effects in fathers and other family members and caregivers. While our analyses were restricted to mother’s voice, empirical evidence suggests that fathers and other caregivers make similar acoustical modifications when speaking with young children^41^, and we hypothesize that reduced pitch height would be evident for these other important vocal sources during later stages of childhood and adolescence.

An interesting question that arises from our findings is: at what age does the height of mother’s pitch during child-directed speech reach the pitch height observed during adult-directed speech. Extrapolating from our results (Fig. 1A) and assuming an adult-directed pitch height (175 Hz) based on data from the ManyBabies Consortium study^42^, our results suggest that around 17.5 years, which is close to the end of adolescence and onset of young adulthood (~ 19 years)^43^, the height of mother’s pitch might reach the pitch height during adult-directed speech. The exact age when pitch height of mother’s voice reaches the level of adult-directed speech remains to be determined in future work.

Parents and caregivers alter their vocal characteristics when speaking with children. These alterations are likely a means of enhancing their communication and connection to their child, and here we provide evidence that mothers continue to make alterations to their voice when speaking with their child across a wide range of child and adolescent development. It is intriguing to speculate about the potential function that changes in maternal vocal pitch height could serve during later stages of childhood and adolescent development. A function that is frequently considered in the context of increased pitch height in child-directed speech is capturing and maintaining the child’s attention^23–25^. Previous studies have observed a decrease in mother’s pitch height that starts when the child is around 4 months of age. This pitch height decrease in early childhood has been interpreted as a shift in mother–child interaction from a face-to-face setting in the first months of a child’s life, which relies on the use of heightened vocal pitch to facilitate capturing and maintaining the attention of the child, towards an actor-observer setting, in which reduced attention-capturing and maintenance by the mother encourages her child to actively explore their immediate environment^20^. However, the social world changes dramatically for older children and adolescents relative to younger children^44^, and the parenting goals during this later phase of child-rearing evolve to meet their child’s needs. A primary goal for parents during adolescence is to prepare their child for more adult-like communication that they will be required to engage in as they approach and enter adulthood. We therefore hypothesize that mothers of older children and adolescents continue to decrease the pitch of their voice as a means of gradually adapting to the nuances of more adult-like communication that they are increasingly required to engage in as they progress towards adulthood. Increased exposure with vocal cues present in adult social interactions may familiarize a child to direct her attention to subtleties in vocal inflections and the content of the speech itself. Accordingly, we posit that reductions in parents’ vocal pitch serves multiple, distinct functions across child development, from encouraging young children to explore their physical environment^20^ to preparing adolescents for adult-like communication.

Here, we investigated the association of mother’s vocal signature with their child’s age in the context of typical development. An important area of research involves investigating the dynamics of mother–child vocal communication in the context of atypical development, including children with neurodevelopmental disorders such as autism spectrum disorder (ASD). Importantly, multiple lines of research provide evidence of aberrant speech production in parents of children with neurodevelopmental disorders. First, it has been demonstrated that many non-diagnosed parents of children with ASD show milder, subclinical traits that qualitatively resemble the clinically defining features of ASD^45,46^. This phenomenon is referred to as the “broad autism phenotype” and reflects the genetic basis of the disorder^47^. Intriguingly, non-diagnosed parents of children with ASD have been shown to use atypical speech^48^, and acoustical analyses of voice samples of non-diagnosed parents reveal a slower speech rate compared to parents of typically developing children^49^. Moreover, previous studies of child-directed speech have shown acoustical differences in mother’s voice in the context of a variety of other disorders, including maternal depression^50,51^, hearing impairments in children^52^, as well as dyslexia^53^. Given these reports of aberrant speech production in parents of children with ASD and other clinical pediatric clinical populations, an important research question is whether mothers of children with neurodevelopmental disorders struggle to provide age and developmentally appropriate modulations to their voice, such as pitch height decreases shown in the current study. Moreover, it is unknown if atypical mother’s voice production is linked to established voice processing deficits in their children^54–56^.

Results from our study show that there was no statistically significant effect of mother’s age on mother’s pitch height. Previous studies have shown that women’s vocal pitch reduces as a function of age, however details from this literature reveal that pitch does not tend to change significantly across the child- and adolescent-raising years, and that the effect of age on vocal pitch is strongest for women over 60 years old^29,30,57^. Given that only one mother included in our study was over 60 years old, our findings are consistent with the literature on age-related changes in woman’s pitch height.

Vocal samples used in this study were collected for a brain imaging study examining the neural processing of mother’s voice in children^5,32^, and therefore adult-directed vocal samples were not collected. Further studies building on this work may also examine mother’s voice in the context of adult-directed vocal samples, as well as non-mother women’s voice. Furthermore, the vocal recording methods used in our study, which included the use of nonsense words while pretending they were speaking to their child, provided tight experimental control instead of a more naturalistic recording with greater ecological validity. It remains a possibility that the recording methods used in our study affected the variability in acoustical features of mother’s voice. Thus, an exciting avenue for future studies will be to examine whether acoustical features associated with child-directed speech show stronger relationships with child’s age when using more naturalistic vocal recordings that may allow for more natural variation in these features.

In conclusion, we have shown that pitch height of child-directed mother’s voice continues to undergo protracted changes during late childhood and adolescence, and this effect is independent of mother’s age. This finding is in contrast to other acoustical features frequently associated with child-directed speech which do not show the same association when pitch height is controlled for and which were not predictive of age. The decrease in maternal vocal pitch may function to gradually prepare their child for the social cues they will experience as adults and independent members of society.

## Supplementary Information

Supplementary Figure S1.

## Data Availability

Datasets used in the current study are available from the corresponding authors upon reasonable request.
