# Supplementary material for: Mothers adapt their voice during children’s adolescent development
Source: Sci Rep. 2022 Jan 19;12:951. doi: 10.1038/s41598-022-04863-2 (PMC8770681; doi:10.1038/s41598-022-04863-2)
Supplement: Supplementary file 1 — Supplementary Figure S1. [file 41598_2022_4863_MOESM1_ESM.pdf]

Supplemental material

Mothers adapt their voice during children's adolescent development

Simon Leipold[1], Daniel A. Abrams[1], Vinod Menon[1,2,3]

[1] Department of Psychiatry and Behavioral Sciences

[2] Department of Neurology and Neurological Sciences

[3] Stanford Neurosciences Institute

Stanford University

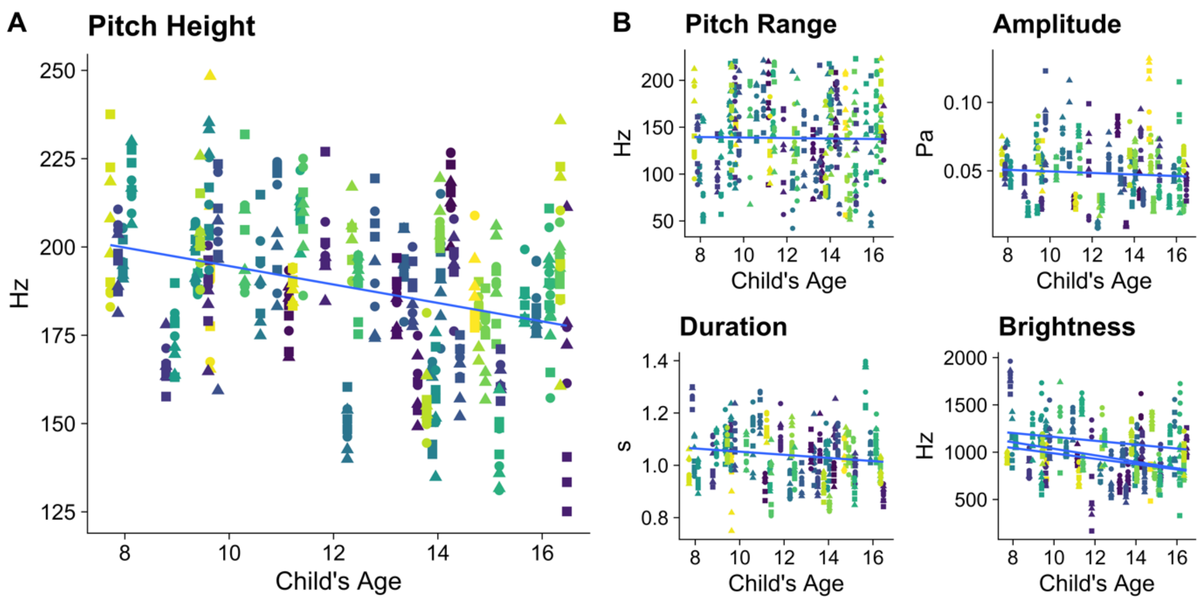

Figure S1.

(A) Maternal pitch height was negatively associated with the child's age when considering variance across nine different voice samples per mother (β_{std} = -0.32, p = 0.005). **(B)** Brightness of mother's voice, an acoustical feature which is correlated with pitch height, also showed a negative association with child's age (β_{std} = -0.26, p = 0.02), however, including an effect of nonsense word resulted in a non-significant association between brightness and child's age. No further acoustical feature of mother's voice showed a statistically significant association with the child's age. Dots of the same color represent values of a single mother. Dots of the same shape represent the same nonsense words ("teebudishawlt", "keebudishawlt", or "peebudishawlt"). For brightness, a timbral feature, an effect of nonsense word was included, which is represented in separate regression lines per nonsense word.
